# Targeted proteomic assays for quantitation of proteins identified by proteogenomic analysis of ovarian cancer

**DOI:** 10.1038/sdata.2017.91

**Published:** 2017-07-19

**Authors:** Ehwang Song, Yuqian Gao, Chaochao Wu, Tujin Shi, Song Nie, Thomas L. Fillmore, Athena A. Schepmoes, Marina A. Gritsenko, Wei-Jun Qian, Richard D. Smith, Karin D. Rodland, Tao Liu

**Affiliations:** 1 Biological Sciences Division, Richland, WA 99354, USA; 2 Environmental Molecular Sciences Laboratory, Pacific Northwest National Laboratory, Richland, WA 99354, USA.

**Keywords:** Ovarian cancer, Proteomic analysis, Biomarkers

## Abstract

Mass spectrometry (MS) based targeted proteomic methods such as selected reaction monitoring (SRM) are emerging as a promising tool for verification of candidate proteins in biological and biomedical applications. The Clinical Proteomic Tumor Analysis Consortium (CPTAC) of the National Cancer Institute has investigated the standardization and analytical validation of the SRM assays and demonstrated robust analytical performance on different instruments across different laboratories. An Assay Portal has also been established by CPTAC to provide the research community a resource consisting of large sets of targeted MS-based assays, and a depository to share assays publicly. Herein, we report the development of 98 SRM assays that have been thoroughly characterized according to the CPTAC Assay Characterization Guidance Document; 37 of these passed all five experimental tests. The assays cover 70 proteins previously identified at the protein level in ovarian tumors. The experiments, methods and results for characterizing these SRM assays for their MS response, repeatability, selectivity, stability, and endogenous detection are described in detail. Data are available via PeptideAtlas, Panorama and the CPTAC Assay Portal.

## Background & Summary

Mass spectrometry (MS) based proteomics has increasingly been considered as an indispensable technology for biological and biomedical research. The unbiased, ‘discovery’ proteomics analysis (e.g., ‘shotgun’ proteomics) can now provide genome-scale coverage and quantification of both proteins^1,2,3^ and posttranslational modifications (PTMs) using extensive fractionation and stable isotope labeling^4,5^. However, verification of these discovery findings requires higher specificity, higher precision and accuracy in quantification and higher sample throughput, while maintaining the sensitivity at the same level, if not higher^6,7^. This is especially the case for studies such as biomarker development^8^.

Targeted proteomic assays such as selected reaction monitoring (SRM; also referred as multiple reaction monitoring, MRM) are an ideal choice for preclinical verification studies^9,10^. SRM has been widely used for sensitive quantification of proteins, mostly using triple quadrupole (QQQ) MS instruments^11,12^. Typically, multiple ‘transitions’, the specific pairs of the precursor and fragment ions, are selected and used for a given peptide for increased specificity. The quantification is typically performed by comparing the peak areas of transitions to those of the internal standards such as heavy isotope-labeled synthetic peptides^13^, providing high specificity as well as high precision and accuracy in quantification^12,14^. Moreover, compared to antibody-based assays, SRM assays are easy to multiplex, are able to distinguish modified and unmodified forms of the protein, and can be developed with very high success rates, significantly shorter lead time and significantly lower cost^11,15,16,17,18,19^.

SRM has also been recently evaluated and demonstrated in several systematic consortial studies as a highly reproducible and transferrable analytical platform for targeted proteomics analysis^14,20,21^. For example, the Clinical Proteomic Tumor Analysis Consortium (CPTAC) of the National Cancer Institute (NCI) has investigated the standardization and analytical validation of targeted protein assays in intra- and inter-lab settings, and has demonstrated robust analytical performances of SRM assays on different instruments across different laboratories^14,22,23,24^. CPTAC has also recently established an Assay Portal (http://assays.cancer.gov/) to provide the research community a resource of large set of CPTAC-developed targeted MS-based assays, as well as a place for researchers outside of CPTAC to share their assays publicly, if the CPTAC assay characterization guidelines (https://assays.cancer.gov/guidance-document/) are met^25^. A guidance document for proper characterization and handling of peptide standards has also been recently established by CPTAC^26^. The availability of the guidance documents and knowledge sharing resources will enable and facilitate highly reproducible, collaborative targeted proteomics in the scientific community to overcome the throughput bottleneck in studies such as biomarker development where large-scale verification efforts are needed.

Herein, we report 98 LC-SRM assays that have been thoroughly characterized by all the 5 experiments described in the CPTAC Assay Characterization Guidance Document; the assays were chosen primarily based on prior observations at the protein level in high grade serous ovarian cancer samples^27^. We also demonstrate the pipeline for assay development and analytical validation of these SRM assays, as well as the distribution of data as a community resource. Corresponding SRM data are stored on PeptideAtlas (Data Citation 1), Panorama (https://panoramaweb.org/labkey/project/Panorama Public/2017/ CPTAC_MRM_Assay_Ovarian_Tissue_PNNL.url), and the CPTAC Assay Portal.

All the 98 SRM assays haven been thoroughly characterized according to CPTAC Assay Experiments 1 to 4 to establish the required confidence in sensitivity, specificity and repeatability; 37 assays were able to provide direct detection of endogenous peptides in an ovarian tumor sample unrelated to the previously analyzed samples, as required in Experiment 5. In a separate experiment, 28 additional endogenous peptides were detected in the same matrix using a higher sensitivity PRISM-SRM (high-Pressure, high-Resolution separations with Intelligent Selection and Multiplexing-SRM) method with front-end chromatography-based enrichment^15^.

These SRM assays are suitable for Tier 2 analysis as defined by the CPTAC ‘fit-for-purpose’ approach^28^. Compared to Tier 1 assays which aim at providing clinically executable information, Tier 2 assays are typically developed for basic research purposes, e.g., to measure changes in the abundance of specific proteins, peptides, or modifications selected on the basis of association with specific phenotypes such as disease type or stage, or to measure response to treatment in a preclinical setting. Tier 2 assays can be used to verify tens to hundreds of candidate proteins identified from discovery studies or the curated literature in independent sample sets, as a first step in biomarker verification or confirmation.

## Methods

### Summary

**Figure 1:**
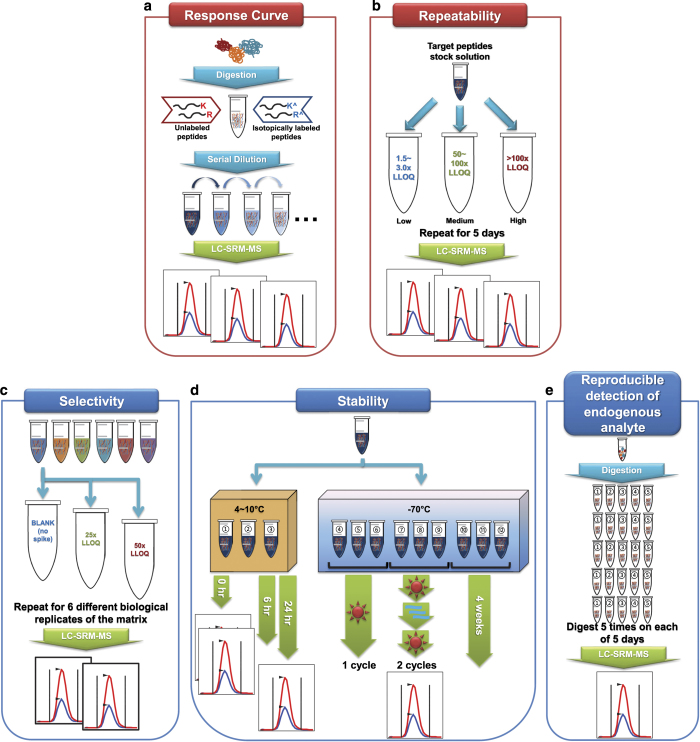
Summary of the five experiments for characterizing the targeted MS assays as recommended by the CPTAC Assay Portal. Experiments 1 and 2 are required for uploading the assays to the Assay Portal while Experiments 3 to 5 are optional. (**a**) Experiment 1 is designated to create a response curve using multiple points of various concentrations of the target peptides. (**b**) Experiment 2 determines the repeatability of assays at 3 different concentration points over 5 days. This mini-validation evaluates inter- and intra-day variability. (**c**) Experiment 3 studies the selectivity (parallelism) of the assays in six different biological replicates of the same matrix type. (**d**) Experiment 4 is designed to check the stability of the target peptides at different storage time and conditions. (**e**) Experiment 5 applies the overall workflow developed in the previous experiments to the detection of the endogenous peptides in the biological samples.

The CPTAC Assay Portal describes five different experiments in the Assay Characterization Guidance Document for characterizing targeted MS based assays as shown in Fig. 1 and the bulleted list below. Each experiment aims at evaluation of different analytical properties of assays. Experiment 1 is to create response/calibration curve for target peptides, assessing different analytical properties including limit of detection (LOD) and limit of quantitation (LOQ). Experiment 2 is to evaluate the repeatability using the LOQ value obtained from the Experiment 1. Experiments 1 and 2 are required for any assay to be uploaded to the CPTAC Assay Portal. Experiment 3 is to study the selectivity of heavy isotope-labeled internal standards spiked into 6 different biological matrices. Experiment 4 is to investigate the stability of target peptides upon 6 different storage or freeze-thaw cycles. Experiment 5 is to assess the overall reproducibility and the ability to detect endogenous analytes for the assays built from this work flow. The experimental procedures used in our SRM experiments are described in details below.

- Experiment 1: Response curve (required for upload).
- Experiment 2: Mini-validation of repeatability (required for upload).
- Experiment 3: Selectivity.
- Experiment 4: Stability.
- Experiment 5: Reproducible detection of endogenous analytes.

### Reagents and samples

Methanol (MeOH), HPLC grade acetonitrile (ACN), and HPLC grade water were obtained from Fisher Scientific (Fair Lawn, NJ). Formic acid was purchased from Agilent Technologies (Santa Clara, CA). Dithiothreitol (DTT), iodoacetamide (IAA), urea, trifluoroacetic acid (TFA), phosphatase inhibitor cocktail 2 (P5726) and cocktail 3 (P0044) were purchased from Sigma-Aldrich (St Louis, MO). Protease inhibitor cOmplete ULTRA Tablet was purchased from Roche (Mannheim, Germany). Trypsin was obtained from Affymetrix (Santa Clara, CA). Discovery DSC-18 SPE cartridges (C18 bonding, 50 mg1 ml^−1^) was purchased from Supelco (Bellefonte, PA).

For the first batch of 39 assays (see Table 1), both the crude heavy stable isotope-labeled peptides and sequence matched pure light versions were synthesized by Thermo Fisher Scientific (Waltham, MA); pure light and pure heavy peptides used for the second batch of 59 assays were purchased from New England Peptides (Gardner, MA). The pure synthetic peptides were >95% in purity by HPLC-UV and quantified by amino acid analysis (AAA) by the vendors. The heavy peptides incorporated a fully atom labeled ^13^C and ^15^N isotope at the C-terminal lysine (K) or arginine (R) position of each (tryptic) peptide, resulting in a mass shift of +8 or +10 Da, respectively. Sequence matched pure light versions were spiked at a constant concentration as internal standards (IS). Aliquots of the peptides were stored in 5% acetonitrile/0.1% formic acid at −80 °C until use. Tryptic digests of proteins extracted from de-identified flash-frozen ovarian tumor tissues were used as matrices in corresponding experiments. All experimental procedures were approved by the human subjects Institutional Review Board of Pacific Northwest National Laboratory in accordance with federal regulations.

### Tissue sample preparation

Lysis buffer was composed of 8 M urea, 75 mM NaCl in 100 mM NH_4_HCO_3_ (pH 7.8), 10 mM NaF, phosphatase inhibitor cocktail 2 and cocktail 3, and cOmplete ULTRA Tablet (1/2 tablet per 5 ml). Ovarian tumor tissue samples and the lysis buffer were chilled on ice. After adding 500 μl of the lysis buffer to the tissue samples, the tissues were homogenized on an ice block for more than 1 min until the tissue samples were thoroughly mixed. The samples were then vortexed for 3 min and sonicated in a sonication bath for 3 min.

Prior to tryptic digestion, protein concentration was determined by BCA protein assay (ThermoFisher Pierce, Rockford, IL). Sufficient amount of the stock solution of 500 mM DTT was added to the samples to make a final concentration of 5 mM followed by incubation at 37 °C for 1 h with shaking at a speed of 1,200 r.p.m. Next, sufficient amount of the stock solution of 400 mM IAA was added to make a final concentration of 10 mM followed by incubation at room temperature for 1 h in the dark with shaking at a speed of 1,200 r.p.m. After the samples were diluted two times, 1 M CaCl_2_ was added to the samples to a final concentration of 1 mM. A 20 μl-aliquot of 50 mM NH_4_HCO_3_ was added to a vial of 20 μg of trypsin followed by incubation at 37 °C for 10 min to activate enzyme. Trypsin was added to the samples at a ratio of 1:50 w/w of enzyme: protein followed by incubation at 37 °C for 4 h with shaking at a speed of 700 r.p.m. After digestion, the samples were diluted 4 times and acidified with TFA to a final pH of 2–2.5 (a final concentration of 0.5% TFA). The samples were preceded to SPE clean-up.

The samples were centrifuged at a speed of 4,000×g for 10 min. The DSC-18 SPE column was prewashed with 3 ml of MeOH and then with 2 ml of 0.1% TFA. The digest samples were slowly added through the column. The columns were then washed with 4 ml of 5% ACN with 0.1% TFA. Finally, the digest peptides were eluted with 1 ml of 80% ACN/0.1% TFA. Another BCA protein assay was performed to obtain a final concentration of peptides and a yield of tryptic digestion. The peptide recovery after digestion and SPE clean-up was about 55~60%.

### Selection and optimization of SRM parameters of target peptides

Peptide selection is a critical step for developing sensitive, selective and reliable SRM assays. The target peptide sequences of the candidate proteins were selected based on public data repositories, previous publications, and our own unpublished data. Several selection criteria that have been previously discussed were considered with additional empirical practices^9,28,29,30^. In general, the target assays were based on tryptic peptides ranging from 8 to 25 amino acids in length. The peptides were required to be fully tryptic without K/R.P-, -R.RH-, -DK.D, or -RRR- at C-terminus^29^. Also, possible modifications on peptides were typically excluded for SRM assay development to ensure reliable quantification^9,28,30^. The modifications included PTMs or artificial modifications such as oxidation on Met, deamidation on Asn followed by Gly, or pyroglutamic acid formation on N-terminal Gln. Peptide detectability was searched through several databases including Global Proteome Database^31^ (GPMDB) and PeptideAtlas^32^, online depositories of identified peptides that have been previously observed in MS/MS analyses. For peptides that are not in the databases, several computational tools such as CONSeQuence^33^ and Peptide Prediction with Abundance^34^ (PPA) were used to predict the likelihood of detectable peptides. To check protein sequence homology, MS-Homology (http://prospector.ucsf.edu/prospector/cgi-bin/msform.cgi?form=mshomology) or UniProt (http://www.uniprot.org/) were searched. According to these criteria, five candidate peptides were initially selected for each target protein and their crude peptides were purchased for optimization of SRM parameters and to check LC and MS responses. For some of the proteins, fewer than 3 candidate peptides were selected due to the small size of proteins hence the limited number of possible tryptic peptides.

Prior to SRM experiments, selection of precursor and product ions *m/z* values (transitions) and collision energies of transitions were optimized by direct infusion to QQQ MS. Target peptides and a list of transitions were loaded and selected from a Skyline file. These lists were used to monitor the behavior during direct infusion on QQQ MS and the four to six best performing transitions were selected. These selected transitions of all target peptides were then assessed by LC-SRM to check the response and interference in an ovarian tumor digest. The number of assays were finalized to two peptides per protein with three to four best performing transitions. Simultaneously, the pure light peptides were synthesized and purchased for the analyses in the five assay characterization experiments. The final developed SRM assays will be further described and discussed in the Usage Notes section.

### Experiment 1: Response curve

The purpose of Experiment 1 is to characterize the linear dynamic range and determine the lower limits of quantification (LLOQ) and detection (LLOD) of the peptide assays using their response curves (Fig. 1a). The LLOD and LLOQ of each assay were calculated at three scenarios: blank only, blank plus low concentration point, and relative standard deviation (RSD) limit (15%). The specific calculation is described in details in Technical Validation below. Crude heavy peptides and pure light peptides were used for the first batch of 39 assays. The concentration of crude heavy peptides was determined from heavy/light ratios obtained from the light-heavy peptide mixing experiment and known concentration of the pure light peptides. Both pure light and pure heavy peptides were used for the second batch of 59 assays.

For the first 39 assays, the stock of crude heavy peptides was serially diluted with 0.25 μgμl^−1^ of tissue digest with the following dilution factors: 1, 10, 100, 1,000, 10,000, 20,000, 50,000, 100,000 and 200,000. A 2 μl-aliquot of the heavy peptide mixtures at different concentrations was then added to 36 μl of the digested tissue matrix. A separate sample, namely blank, was made without the heavy peptides by adding 4 μl-aliquot of buffer A into 72 μl of the digested tissue matrix. A 2 μl-aliquot of light IS peptide mixture was further added to different concentrations of the heavy peptides to make a final volume of 40 μl while 4 μl of light IS peptide mixture was added to the blank sample to make a final volume of 80 μl. For the other 59 assays, the stock of pure heavy peptides was serially diluted with 0.1 μgμl^−1^ of tissue digest matrix with the following dilution factors: 1, 2, 4, 10, 20, 100, 200, 1,000, 2,000, 10,000, 20,000, 40,000, 100,000 and 200,000. A 4 μl-aliquot of different concentration of the heavy peptide mixture was then added to 34 μl of the digested tissue matrix. A 2 μl-aliquot of light IS peptide mixture stock (50 fmolμl^−1^) was further added to different concentration of the heavy peptides to make a final volume of 40 μl. A separate blank sample was prepared by adding 2 μl-aliquot of light IS peptide mixture stock to 72 μl of the digested tissue matrix followed by an addition of 4 μl of buffer A. The on-column amounts of the peptides at different concentration points are detailed in Experimental Information worksheet 1a for batch 1 assays and 1b for batch 2 assays (Data Citation 2). All of the samples were prepared in Waters LCGC certified glass vial (Waters, Milford, MA). The samples were shaken on Eppendorf ThermoMixer (Eppendorf, Hamburg, Germany) at a speed of 800 r.p.m. and at a temperature of 4 °C for 10 min. LC-SRM were run with an order of the blank (>9 runs), low concentration to high concentration in triplicates.

### Experiment 2: Mini-validation of repeatability

Two terminologies are commonly used to examine analytical variability. Repeatability is the variation in the successive measurements on the same sample, using the same system and operator, and under the same conditions over a short period time^35,36^. Therefore, the variation in run-to-run measurements can be estimated to check the repeatability of the analytical technique. Reproducibility is the variation of the measurements observed when the system, operator, conditions, or time of measurements is changed^35,36^. For example, the variation between two instruments or results from two different laboratories can be used to assess the reproducibility of the data. The purpose of Experiment 2 is to provide data on the repeatability of measurement over 5 consecutive days (Fig. 1b). This mini-validation of repeatability of assay measurements mimics an actual experiment analyzed spanning over many days. Two types of measurement variability can be calculated: intra-assay and inter-assay. Three different concentration points were defined by the CPTAC Assay Characterization Guidance Document: low concentration (1.5–3×LLOQ), medium concentration (50–100×LLOQ) and high concentration (>100×LLOQ). Each concentration point was analyzed in triplicates (or more) over 5 days in which different days are defined as two calendar days more than 16 h apart. Injections of these three concentrations of the samples were randomized since in the case of actual biological samples unknown concentrations are measured. Blank injections are inserted to reduce carryover from high or medium to low concentration of the sample.

The calculation of the LLOQ used for Experiment 2 is described in Technical Validation below. In our assays, the crude heavy peptides were spiked into tissue digest matrix with concentrations at 1.5–3×LLOQ for low concentration, 50–100×LLOQ for medium concentration, and >100×LLOQ for high concentration. Since not all of the peptides showed the same LLOQ values, ranges of concentrations were used to cover all the full range of target peptides for the experiment. The peptide stocks used in Experiment 1 were serially diluted with tissue digest matrix to create the different concentration points as described above. A 2 μl-aliquot of each concentration points of the heavy peptides was added to 36 μl of tissue digest matrix (the same matrix used in Experiment 1). A 2 μl-aliquot of light IS peptide mixture was added to each sample to make a final volume of 40 μl. The on-column amounts of peptides at different concentration point are listed in Experimental Information worksheet 1c (Data Citation 2). The samples were analyzed in triplicates in randomized order on 5 consecutive days.

### Experiment 3: Selectivity

Experiment 3 determines if the response is similar in 6 different biological replicates of the matrix (so called parallelism) (Fig. 1c). Here, the different biological replicates refer to the source of the matrix (i.e., different patients or conditions), rather than the type of matrix (e.g., serum, plasma, or urine). For example for serum samples, six different biological replicates would be serum samples originating from six different individuals. For cell lines, they might be six different cell lines or the same cell line treated with six different perturbations. In the case of tumors, six different tumors or three different tumors and three paired adjacent normal tissue samples could be used to represent six different biological replicates.

Six different ovarian tumor tissue samples were prepared as representative matrices for Experiment 3. The peptide stocks used in Experiment 2 were used to create three different concentration points: zero spiking in, medium concentration as defined in Experiment 2 and 1/2 of the medium concentration. A 2 μl-aliquot of each concentration points of the heavy peptides was added to 36 μl of tissue digest matrix. A 2 μl-aliquot of light IS peptide mixture was added to each sample to make a final volume of 40 μl. A total of 18 prepared samples were analyzed in a randomized order on the same day in duplicate.

### Experiment 4: Stability

The purpose of Experiment 4 is to assess the variation of the measurements after different sample storage times and conditions (Fig. 1d). The matrix for this experiment was spiked with the medium concentration as defined in Experiment 2. The sample was aliquoted into 12 vials, of which three were put in an auto-sampler (~4 °C) of LC system. These samples were immediately injected in duplicate after preparation. The same samples were analyzed again once after at least 6 h and once after at least 24 h in the auto-sampler. The other 9 vials were stored in a freezer at −70 °C. Three of them (the forth to sixth samples) were thawed at room temperature for an hour and analyzed by LC-SRM. These three samples passed through 1 freeze-thaw cycle. Another three samples (the seventh to ninth samples) were thawed at room temperature, put back to the freezer at −70 °C, and thawed at room temperature again for SRM analysis. These three samples passed through 2 freeze-thaw cycles. The last three samples (the tenth to twelfth samples) were analyzed after four weeks of storage at −70 °C.

### Experiment 5: Reproducible detection of endogenous analytes

Experiment 5 assesses the overall reproducibility of the assay workflow built from the previous experiments, including both sample preparation and LC-MS analysis (Fig. 1e), as well as the ability of the assays to detect the endogenous analytes using heavy peptides as standards. To do this, portions of the same ovarian tumor tissue sample were digested 5 times on each of 5 days. A 2 μl-aliquot of heavy peptide mixture was added to each sample to make a final volume of 40 μl. The five digests were analyzed each day. In a separate experiment (not part of Experiment 5), a higher sensitivity method, PRISM-SRM with front-end chromatography-based enrichment was performed for the endogenous peptides that could not be quantified by standard LC-SRM analyses, to check their detectability in the ovarian tumor matrix used in Experiment 5. Also, to evaluate the quantification accuracy of these SRM assays, ELISA assays were performed to measure endogenous level of PCNA and IL18 proteins.

### PRISM-SRM assay for the peptides not endogenously quantified in experiment 5

Different volumes equivalent to 10 μg of peptides from 5 samples of the Experiment 5 were pooled together. The stock of heavy peptides was spiked in 35 μg of the pooled digested tissue matrix to 4 fmolμl^−1^ in a final sample volume of 50 μl. The sample was then fractionated by PRISM system into 96 fractions as described in details in our previous study^15^. Briefly, the target fractions were separated by high-resolution capillary RPLC using high pH mobile phases. The eluent from the capillary RPLC column at a flow rate of 2.2 μlmin^−1^ was split into two flow streams via a Tee union (the split ratio of flow rates is 1:10). The small portion was used for online SRM monitoring of internal standard peptides and the large portion was deposited into a 96-well plate every minute automatically during a 100-min LC run. The specific target peptide fractions were selected with intelligent selection (*i*Selection) for downstream regular LC-SRM analysis.

### ELISA and SRM assays for PCNA and IL-18 proteins

PCNA and IL-18 proteins ELISA kits (product code: ab196270 and ab215539) were purchased from Abcam (Cambridge, MA). The ELISA assay was performed according to the manufacturer’s instructions. The samples were diluted to 0.1 μgμl^−1^ of total protein concentration based on BCA protein assay and measured by the ELISA assays in triplicates. For SRM assays, the stock of heavy peptides was spiked into 6 ovarian cancer tissue digests (2 benign controls, 1 stage I, and 3 stage IV tumors; see Supplementary Fig. 3). The final concentration of heavy peptides was 10 fmolμl^−1^ in 0.125 μgμl^−1^ of the tissue matrix.

### LC-SRM analysis

LC-SRM analysis was performed on a Waters nanoACQUITY UPLC system (Waters, Milford, MA) interfaced to an TSQ Vantage mass spectrometer (Thermo Scientific, San Jose, CA) equipped with a nano-ESI source. An injection volume of 4 μl was used for the analysis. Separation was then performed using an ACQUITY UPLC Peptide BEH C18 nanoACQUITY column (100 μm id×100 mm, 1.7 μm, 130 Å, Waters, Milford, MA). The mobile phase A was 0.1% formic acid in water and mobile phase B was 0.1% formic acid in acetonitrile. To achieve separation the following flow gradient was used: starting at 0.5% solvent B with a flow rate of 500 nlmin^−1^, ramping of 0.5–5% solvent B for 0–9 min, ramping of 5–8% solvent B for 9–13 min, ramping of 8–10% solvent B for 13–20 min, ramping of 10–12.5% solvent B for 20–26 min, ramping of 12.5–14.5% solvent B for 26–34 min, ramping of 14.5–22% solvent B for 34–48 min, ramping of 22–35% solvent B for 48–52 min, ramping of 35–95% solvent B for 52–54 min, maintaining 95% solvent B for 54–60 min with increasing a flow rate to 750 nlmin^−1^, decreasing 95–10% solvent B from 60–62 min, increasing 10–95% solvent B from 62–64 min, maintaining 95% solvent B for 64–65 min, decreasing 95–0.5% solvent B from 65–67 min, and maintaining 0.5% solvent B from 67–75 min with decreasing a flow rate to 500 nlmin^−1^.

The TSQ Vantage mass spectrometer was operated in positive ion-mode with the ESI voltage set to 2,400 V and a capillary temperature at 325 °C. The SRM experiment was programmed to conduct scheduled SRM assays. The optimum collision energy was set for each transition of the different peptides. The summary of SRM parameters for 98 peptides is described in Experimental Information worksheet 1d (Data Citation 2), noting precursor and production *m/z* values, collision energy, and retention time. Depending on the specific retention time of different peptides, the SRM detection window was set to 5 and 6.5 min for batch 1 and 2 assays, respectively. The cycle time was set to 1 s and the dwell time for each transition was automatically adjusted depending on the number of transitions scanned in the different retention time windows. The collision gas pressure was set to 1.5 mTorr and the tuned S-lens value was used from the current tune method saved in the TSQ Vantage mass spectrometer. The resultant raw data were analyzed by Skyline^37^. Details for evaluations of each experiment and Skyline parameters are described in the following **Data Records** section. Also, the determination of analytical characteristics of the developed SRM assays, including LLOD or LLOQ, are discussed in the **Technical Validation** section.

## Data Records

LC-SRM data were imported to Skyline to quantify peptides (Data Citation 1)^37^. A FASTA file containing targeted proteins was imported in the Skyline document. Necessary information about the targeted proteins include protein name (e.g., sp|P04677|), UniProt/SwissProt protein accession number, protein description, protein gene name, protein species, and protein sequence. The protein information can be found in Document Grid of Skyline document. Separate Skyline files need to be made for different experiments. More detailed guideline about how to create and upload Skyline document can be found on the Skyline website. Corresponding standard operating procedures (SOPs) for each experiment were prepared, describing sample preparation, enzymatic digestion, sample handling, and LC-SRM method parameters; associated metadata was also entered into Panorama and the CPTAC Assay Portal to show the details and assay parameters such as the type of MS instrument and matrix, matrix amount, standard peptide purity, data type, the type of LC system, gradient, SOPs and *etc.*

Specific annotations were needed to demonstrate the results. Under Document Settings in Skyline document, custom annotations were added including **SampleGroup**, **Replicate**, **ISSpike**, **Concentration**, **PeptideConcentration**, **PeptideConcentrationIS**, and **MultiplicatoinFactor**. Annotation **SampleGroup** represents the group of a dataset while **Replicate** shows the order of a group containing replicate injections. Both annotations **ISSpike** and **PeptideConcentrationIS** show the concentration at which the internal standard peptide is spiked in. **PeptideConcentrationIS** is used when IS peptides are spiked in at different concentrations, while **ISSpike** is used when all the IS peptides are spiked in at the same concentration. Similarly, both annotations **PeptideConcentration** and **Concentration** show the concentration at which the target peptide is spiked in. **PeptideConcentration** is used when target peptides are spiked in at different concentration, while **Concentration** is used when target peptides are spiked in at the same concentration. Using an annotation of **MultiplicationFactor**, different concentration was calculated based on multiplier relative to the highest point of the response curve (e.g., 0.1, 0.01, 0.001, etc.). In an experiment of repeatability, an annotation of **PeptideConcentration** refers to a medium concentration point of the curve and that is also the base of **MultiplicationFactor**. Other annotations were adjusted according to SOPs. Detailed information of above mentioned annotations are shown under Results Grid in Skyline document.

More details on the experimental information, including the list of selected peptides, TSQ parameters, and on column amounts of peptides for Experiments 1 and 2, are available on figshare (Data Citation 2). Evaluated results of the 98 assays from the 5 experiments are also available on figshare (Data Citation 3), according to the CPTAC Assay Portal guideline as described in the following **Technical Validation** section. The peak areas of individual measurements from the 5 experiments can be found on figshare as well (Data Citation 4).

## Technical Validation

The CPTAC Assay Portal document requires using at least 3 transitions for valid quantification of peptides. During the spiking steps, the spiked synthetic peptide volume should be lower than 10% of the final volume of prepared samples across all experiments. Since LLOQ or LLOD values determined from Experiment 1 were used for the rest of experiments, there were other considerations to substantiate the quantification of peptides in Experiment 1. For blanks, more than 9 runs and at least 3 runs for all other concentration points should be acquired. Although the samples with different concentration points were made by serial dilution, the injection should be made from the lowest to the highest concentration samples to reduce carryover. There are three methods to compute LLOD and LLOQ. In the first method, LLOQ is determined by 10 times the s.d. of the blank sample plus the average peak ratio of the blank sample and LLOD is determined by 3 times the s.d. of the blank sample plus the average peak ratio of the blank sample. The method is called ‘blank only’. Another method was to use s.d. of blank sample and the average peak ratio of the sample with the lowest concentration to compute LLOD and LLOQ in similar way (so called ‘blank+low’). For the first two methods, blank samples without spiking peptides in the same matrix were used to provide a better determination of the signal-to-noise of the sample matrix by checking the chemical noise contributions from the matrix^38,39^. Also, blank samples can be used to see if there is any potential for interferences or analyte carryover that may hamper the accurate quantitative analysis in case of unknown samples. This criteria is provided by the guideline document from CPTAC Assay Portal. The third way was to use the variability of the assay at each point of the curve. LLOQ is the lowest concentration at which the variability of the assay is estimated to be 15% CV (coefficient of variation) using interpolation. The method is called ‘RSD limit’. LLOD will be calculated as the LLOQ divided by 3. Linear regression was used to calculate the linearity of the response curve (over any three points of the curve). Since three LLOQ values are slightly different from each other, we chose the highest LLOQ value to report for Experiment 2.

In Experiment 2, the intra-assay and inter-assay variabilities are assessed. For the intra-assay variability, the CV values of triplicate injections at each concentration point were calculated on each of the five days. These determined CV values were averaged to represent the averaged intra-assay variability. For the inter-assay variability, the CV values of the first injection at each concentration point across the five days were calculated. The same step was applied to calculate CV of the second and third injections. These calculated three CV values were averaged. The total CV was calculated as the square root of the sum of squared average CV of intra-assay and squared average CV of inter-assay. Accordingly, the intra-assay variability, the inter-assay variability, and the total variability were reported to the CPTAC Assay Portal. Also, the ratios of transitions used for a total of 45 injections (3 concentration points×3 injections×5 days) were estimated within 30% of the mean.

In Experiment 3, the linear slope of the line for each biological replicate should be within 10% of the mean. Also, the half-medium concentration for each biological replicate should not be more than 10% different than the predicted measurement from the medium concentration spiked samples. As similar to Experiment 2, the ratios of transitions needed to be within 30% of the mean. For Experiment 4, the variability of six different storage conditions should not exceed either the variability determined from Experiment 2 or the variability measured from the time zero analyses. For Experiment 5, the data processing was performed similar to Experiment 2.

## Usage Notes

Three tiers of targeted SRM assays have been described as ‘fit-for-purpose’ validation strategy at a workshop jointly sponsored by the NCI and the National Heart, Lung, and Blood Institute^23^. The SRM assays described herein meet requirements for Tier 2 assays. Tier 2 assays are typically developed to measure changes in the abundance levels of proteins, peptides, or modifications that are associated with specific diseases, disease stage, or response to treatment. Tier 1 assays are designed to provide clinically executable information for medical practitioners or for informing decision-making in the development of drugs for human use. Tier 1 assays require an intense level of characterization and analytical validation for a small number of targets which will be analyzed across hundreds to thousands of samples for clinical validation. In contrast, Tier 2 assays are intended to narrow the field of potential targets by validating tens to hundreds of candidate proteins identified from discovery studies or previous literatures across a small number of samples from an independent sample set. This verification step confirms quantitative changes that were observed in the initial discovery experiment. To accomplish this, Tier 2 assays require high selectivity and repeatability and moderate-to-high sensitivity and reproducibility where multiple validation steps are needed to establish confident and precise assays. Accordingly, the 5 experiments described in this study (which are also available at the CPTAC Assay Portal) aim at providing such analytical validation of the Tier 2 assays for nonclinical applications.

Selection of surrogate peptides for the protein targets is a critical step in SRM assay development and has direct impact on all the performance characteristics of the assays. In this study, several peptide selection criteria were considered in order to provide sensitive and reliable assays as mentioned in the **Methods** section. However, peptide selection is also an empirical procedure that balances ideal attributes of the assays with practical limitations (e.g., the tryptic peptides available from the protein sequence and their MS response). It is therefore difficult to adhere to the peptides satisfying all the criteria. In this study, 4 peptides contain potential deamidation sites (AsnGly in the sequence) due to the peptide sequence availability issue; however their repeatability has been well-demonstrated in Experiments 1–4, hence they are deemed suitable for the intended Tier 2 applications. We did not exclude the 14 Cys-containing peptides from the assay development based on our understanding of the current practice and extensive experience in targeted proteomics, since we find that Cys-containing peptides are in general similar or better in MS response, and carbamidomethylation of the Cys-containing peptides can be achieved at very high efficiency and reproducibility. There are also 4 peptides containing the N-linked glycosylation motif AsnXaaSer/Thr, where Xaa is any amino acid, except Pro, however we have checked the UniProt database and confirmed that no glycosylation was reported on these site in the specific peptides targeted.

Quantification of proteins using SRM assays is typically performed using two or more peptide assays when possible for improved quantitation accuracy. We started with 5 peptides per protein for the initial screening, and kept the 2 best performing peptides per protein during the detailed characterization of the multiplexed assays (i.e., Experiments 1–4). Approximately 30% of the peptides were excluded from the final report because they did not meet certain specific requirement(s) of the CPTAC Assay Portal (e.g., dynamic range, precision, interference), resulting in 42 proteins that were only covered by a single peptide assay (a flow chart is provided in Supplementary Fig. 1 as a summary of the assay development for the 70 protein targets). However, we have provided corresponding information on the additional peptides for the same proteins in Experimental Information worksheet 1e (Data Citation 2). While not optimal, we also note that it is a common practice to quantify proteins based on a single, best-performing well-characterized peptide, especially in the case of highly multiplexed assay panels for measuring lower abundance proteins in complex biological samples where typically only the most responsive peptide is detectable^40,41^. Therefore, we believe these carefully evaluated single-peptide assays are still suitable for Tier 2 applications.

**Figure 2:**
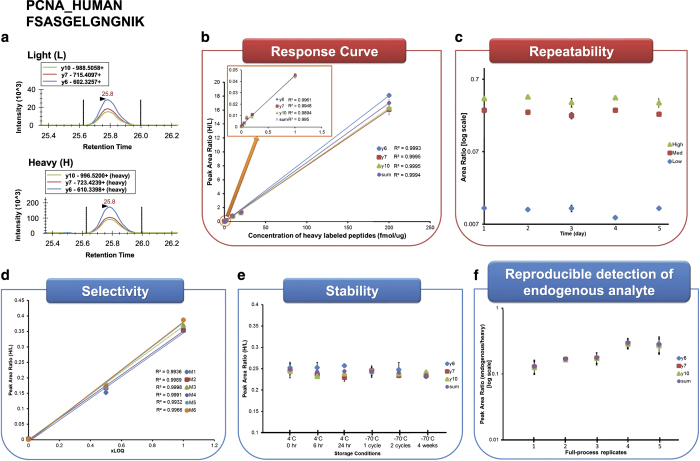
An example of the evaluation results from the five experiments. The data was associated with peptide FSASGELGNGNIK from the proliferating cell nuclear antigen (PCNA) protein. (**a**) A representation of extracted ion chromatograms for light and heavy peptides each with three transitions. (**b**) Peak area ratio of light to heavy peptides for each transition and summation of transitions (sum) was used to plot the linear slope over different concentration points with representative R^2^ values. An inset graph shows the linear slope over the low range of concentration points. (**c**) The measurement variability of this assay was assessed at 3 different concentration points across 5 days. (**d**) Similar to Experiment 1, the linear slope was constructed at three concentration points to assess the influence of matrix differences. It was performed for 6 different biological matrixes (M1 to M6) with representative R2 values. (**e**) From Experiment 4, the stability of heavy peptides can be determined by creating the peak area ratio for each transition and their summed value. This can help the researchers to determine proper handling of peptides. (**f**) When the endogenous peptides were detected, peak area ratios of endogenous to heavy peptides were represented for each transition and their summed value. The reproducible detection can be checked using the 5 full process replicates from the sample preparation to LC-SRM analysis.

The schematic workflow of the 5 experiments is illustrated in Fig. 1 and corresponding results of one example assay are shown in Fig. 2. It is peptide FSASGELGNGNIK originating from the proliferating cell nuclear antigen (PCNA) protein. Figure 2a shows chromatograms of 3 transitions (y6, y7, and y10) of light and heavy peptides with their detected retention times. The CPTAC Assay Portal requires uploading a Skyline document for Experiments 1 (response curve) and 2 (repeatability) and depicts corresponding analytical characteristics as shown in Fig. 2b,c, respectively. In addition, a separate Skyline document is needed to exemplify chromatograms for quantified peptides as shown in Fig. 2a. Thus, at least 3 Skyline documents demonstrating Fig. 2a–c are built and accessible on Panorama and the CPTAC Assay Portal. For Experiment 3, the linear slope and R^2^ values of three different concentration points detected from 6 different biological replicates were assessed as shown in Fig. 2d. Figure 2e depicts the stability of peptides under 6 different storage conditions and freeze-thaw cycles while Fig. 2f exhibits that endogenous peptides (without using light peptides) were detected from 5 full-process replicates over 5 days, indicating the variability likely to be observed in real samples analyzed over multiple days.

The list of the 98 peptides with high-quality SRM assays developed in our laboratory is shown in Table 1. Evaluation data of those assays by the 5 experiments are provided in details in Evaluated Data worksheets (Data Citation 3) according to the CPTAC Assay Portal guideline as described in the **Technical Validatio**n section. The peak areas of individual measurements from the 5 experiments can be found in Peak Areas of Individual Measurements worksheet (Data Citation 4). In this study, the 98 SRM assays were well-characterized by Experiments 1 to 4, while 37 endogenous peptides were quantified by LC-SRM in Experiment 5. For Experiment 1, the LLOD and LLOQ values determined by the ‘blank only’ method are listed as representative data from our study. Three variabilities including intra-assay, inter-assay, and total assay determined by Experiment 2 are also listed along with averaged peak area ratio at three different concentration points. The variability of the linear slopes of each peptide detected from 6 different biological replicates are calculated from Experiment 3. The linear slopes and R^2^ values of three different points from each replicate are also included.

In the case of Experiment 4, the ratios of peak areas at different storage conditions/freeze-thaw cycles are calculated to the ones at the time point zero analyses. Peak areas of the light and heavy peptides were also measured and compared as shown in Supplementary Fig. 2. For the batch 1 assays, the peak areas of light and heavy peptides were similar from time point zero (T0) to 2 freeze-thaw cycles (Thaw2) (Supplementary Fig. 2a,b), while the peak areas for both light and heavy peptides increased in the analysis of the samples after one-month storage (1month). This is coincident with an instrument calibration performed between the Thaw2 and 1month measurements. The data therefore indicate that there is no significant changes in physicochemical properties of the peptides because both peak areas and their ratios of the target peptides did not decrease over time. (Evaluated Data worksheet 2d (Data Citation 3) and Supplementary Fig. 2c). Compared to the T0 analysis, the peak area ratios were ranging from 0.85 to 1.10, from 0.88 to 1.10, from 0.84 to 1.14, from 0.88 to 1.14, and from 0.83 to 1.25 for T6h, T24h, Thaw1, Thaw2, and 1month conditions, respectively. On the other hand, peak areas of light and heavy peptides from batch 2 assays showed a slight decrease (no more than 2-fold) for the T24h time point measurements (Supplementary Fig. 2d,e). We believe that this might be originating from poor spray ionization at the time of the T24h analyses because the peak area ratios were observed to be stable over different conditions, in which both intensities of light and heavy peptides decreased to the same extent. Overall, no significant changes in the assay characteristics of target peptides were observed over different conditions (Evaluated Data worksheet 2d (Data Citation 3) and Supplementary Fig. 2f). Compared to the T0 analysis, the peak area ratios were ranging from 0.89 to 1.15, from 0.83 to 1.19, from 0.91 to 1.22, from 0.91 to 1.06, and from 0.86 to 1.18 for T6h, T24h, Thaw1, Thaw2, and 1month conditions, respectively. Therefore, the similarity in these ratios indicates the stability of the samples throughout this experiment. Averaged CV values were 7.26 and 4.08% for the peak areas and peak area ratios of the 98 SRM assays, respectively.

From Experiment 5, 37 endogenous peptides are quantified by LC-SRM and 28 additional peptides are determined to be endogenously quantifiable in the same matrix of an ovarian tumor digest using PRISM-SRM (Evaluated Data worksheet 2e, Data Citation 3). Here, PRISM-SRM was a single independent study to check the detectability of peptides which were not quantified from the Experiment 5. Therefore, the CV values were not available. A total of 65 peptides appeared to be endogenously quantifiable in the specific tumor tissue matrix tested by us; 33 peptides remain undetected by either LC-SRM or PRISM-SRM, which suggests that corresponding proteins may be expressed at extremely low concentrations in the ovarian tumor sample used or due to lower LC-MS responses, if present at all. It remains to be determined whether those assays can be used in other relevant samples (e.g., ovarian tumors from other patients or cell lines) since 88% of those peptides have been identified in other studies. The total number of observations for those peptides by MS/MS analyses can be found in PeptideAtlas (Evaluated Data worksheet 2e, Data Citation 3). Additionally, relatively higher inter-assay CV values (34% on average) are noted compared to the intra-assay CV values (13.8% on average) for these SRM assays. On different days of preparing the tissue samples, several experimental factors in full-process replicates may cause variation in reproducible detection of endogenous peptides, such as tumor heterogeneity of the tissue sections, and variation in digestion efficiency and/or BCA assay. We believe that most of variations originated from tumor heterogeneity of the tissue sections used in the different days, since the samples were prepared using the same SOPs that have been also used in other Experiments where reproducible results were generated. As expected, higher inter-assay CV values and lower intra-assay CV values were observed, because they were based on the variation on different full-process replicates and injections of the same matrix over different day analyses, respectively.

To evaluate the quantification accuracy, two of the target proteins evaluated in our assays was also measured by ELISA assay. Proliferating cell nuclear antigen (PCNA) protein is known as a member of the so called DNA sliding clamp family which interacts with several proteins involved in DNA replication and repair^42,43^. It has been suggested that PCNA plays an important role in cancer owing to its function in cell proliferation including in ovarian cancer^44,45,46^. The endogenous level of PCNA protein was measured by both ELISA and SRM assays in 6 different ovarian cancer tissue samples. A good correlation was observed for PCNA quantification between ELISA and SRM assays, representing R^2^ value of 0.9627 (2 benign controls, 1 stage I, and 3 stage IV, see Supplementary Fig. 3). Moreover, both assays showed that significant higher levels of PCNA protein were observed in samples from patients with stage IV ovarian cancer compared to the ones with stage I or benign diseases. The data agree well with previous studies indicating that the observed higher concentration of PCNA protein is related to cancer progression, although the number of samples used in this study is rather small^44,47^. IL-18 was also measured by both ELISA and SRM assays in the same set of 6 patient samples, and the ELISA and SRM results showed a moderate R^2^ value of 0.7132 (data not shown). These data demonstrate the robustness of the peptide SRM assays for measuring proteins in complex biological and clinical samples.

More specific SRM parameters of those 98 SRM assays are listed in Experimental Information worksheet 1d (Data Citation 2), including precursor and production *m/z* values, collision energy, and retention time. This table can be easily modified to specific format (e.g., csv) required by a given QQQ mass spectrometer. Therefore, the method may be readily imported for building SRM experiment method, requiring minimal (or no) front-end assay development and enabling immediate application of these assays for SRM based quantitation. Corresponding Skyline files, metadata files and SOPs are available at PeptideAtlas (Data Citation 1). The data can be also found on Panorama (https://panoramaweb.org/labkey/project/PanoramaPublic/2017/CPTAC_MRM_Assay_Ovarian_Tissue_PNNL.url).

These 98 SRM assays covering 70 ovarian cancer related proteins were characterized in tumor tissue matrices, therefore they are suitable for applications such as preclinical verification of candidate proteins for diagnosis, prognosis, or response to treatment, using tumor tissue or biopsy specimens. Given the sample complexity of the tissue, it’s also expected that these assays can be used in cell line based studies without significant changes. However, it’s recommended that the assays be re-evaluated in appropriate matrices if they are to be applied in biofluid-based verification studies. For example, the 22 most abundant proteins in blood plasma/serum make up almost 99% of the total protein mass, as a result these proteins cause a significant ‘masking’ effect for the lower abundance proteins in the plasma/serum sample, if they are not removed by techniques like immunoaffinity depletion; the tremendous complexity of the biofluids may also introduce unexpected interference to certain transitions of the assays. If the interference issues cannot be addressed by using alternative sample processing or separation/enrichment methods, additional peptide(s) from the affected proteins may need to be selected and tested for protein quantification.

## Additional Information

**How to cite this article:** Song, E. *et al.* Targeted proteomic assays for quantitation of proteins identified by proteogenomic analysis of ovarian cancer. *Sci. Data* 4:170091 doi: 10.1038/sdata.2017.91 (2017).

**Publisher’s note:** Springer Nature remains neutral with regard to jurisdictional claims in published maps and institutional affiliations.

## Supplementary Material



Supplementary Information

## Figures and Tables

**Table 1:** List of 98 SRM assays covering 70 proteins (all Cys are carbamidomethylated).

**Batch**	**Protein**	**Peptide**
1	ANR27_HUMAN	LNDPSVVTPFSR
1	ANR27_HUMAN	LYDLPDEPFTR
1	ANX11_HUMAN	DAQELYAAGENR
1	ASGR2_HUMAN	ADHDALLFHLK
1	BAT1_HUMAN	VLSYISVR
1	BAT1_HUMAN	LGSYVQNIFTAAK
1	C3AR_HUMAN	STHC PSNNVISER
1	CD79A_HUMAN	SHGGIYVC R
1	CDK1_HUMAN	LESEEEGVPSTAIR
1	CEAM1_HUMAN	TIIVTELSPVVAKPQIK
1	CO3A1_HUMAN	GPVGPSGPPGK
1	COBA1_HUMAN	GEVGQIGPR
1	COBA1_HUMAN	GFDGLPGLPGDK
1	DLGP5_HUMAN	DVNIPTLEGR
1	ETS1_HUMAN	YENDYPSVILR
1	EVI2B_HUMAN	STPGFILDTTSNK
1	FCERG_HUMAN	AAITSYEK
1	FCG2B_HUMAN	VTFFQNGK
1	GPR20_HUMAN	YLAIVRPEGSR
1	KSYK_HUMAN	LIATTAHEK
1	LYL1_HUMAN	SQPAPPADPDGSPGGAARPIK
1	LYL1_HUMAN	DQAAALAAGPTPPGPR
1	MCM10_HUMAN	GTNLIIQETR
1	MCM10_HUMAN	IGGETLLPR
1	MENTO_HUMAN	LLIVQDASER
1	MENTO_HUMAN	VLILAYAVC R
1	OSGI1_HUMAN	AVDDPGLVFNQLPK
1	PEPD_HUMAN	FEVNNTILHPEIVEC R
1	PEPD_HUMAN	VPLALFALNR
1	PESC_HUMAN	LAALSASLAR
1	PESC_HUMAN	FLLHEPIVNK
1	PK3CD_HUMAN	GELLNPTGTVR
1	PTPRC_HUMAN	LFLAEFQSIPR
1	SFTA1_HUMAN	NPEENEAIASFVK
1	SYTC_HUMAN	GFQEVVTPNIFNSR
1	SYTC_HUMAN	AELNPWPEYIYTR
1	TLE1_HUMAN	FTIPESLDR
1	TOP2A_HUMAN	TLAVSGLGVVGR
1	ZN592_HUMAN	AAPLIVEVFNK
2	ADCY9_HUMAN	TDAHFVDVIK
2	ADCY9_HUMAN	VIPQHQLSISPDIR
2	ARHG1_HUMAN	ELVPPDTLHSLPK
2	AURKB_HUMAN	SNVQPTAAPGQK
2	AURKB_HUMAN	LPLAQVSAHPWVR
2	B2MG_HUMAN	VEHSDLSFSK
2	B3GN8_HUMAN	NLLLVRPLGPQASIR
2	CACO1_HUMAN	GAQELAASSQQK
2	CACO1_HUMAN	LQLEGQVTELR
2	CCNB1_HUMAN	TALGDIGNK
2	CDN1B_HUMAN	VSNGSPSLER
2	CDN1B_HUMAN	NLFGPVDHEELTR
2	CO3_HUMAN	TIYTPGSTVLYR
2	EP300_HUMAN	LGTFLENR
2	ESR1_HUMAN	LLFAPNLLLDR
2	FOXM1_HUMAN	VLLAEEGIAPLSSAGPGK
2	GNAI1_HUMAN	DSGVQAC FNR
2	GNAI1_HUMAN	IAQPNYIPTQQDVLR
2	GNAI3_HUMAN	IDFGEAAR
2	GNAI3_HUMAN	ISQSNYIPTQQDVLR
2	GRB10_HUMAN	SQQDPAGPGLPAQSDR
2	HDAC1_HUMAN	YYAVNYPLR
2	IKKA_HUMAN	LGTGGFGNVC LYQHR
2	IKKA_HUMAN	VWAEAVHYVSGLK
2	IL18_HUMAN	ISTLSC ENK
2	IL18_HUMAN	SDIIFFQR
2	JAK1_HUMAN	QLASALSYLEDK
2	JAK2_HUMAN	LSDPGISITVLPK
2	JUN_HUMAN	NSDLLTSPDVGLLK
2	MPIP2_HUMAN	LLGHSPVLR
2	MPIP2_HUMAN	TAVNLPLER
2	NCOA1_HUMAN	LVQGGGLDVLSER
2	NCOA3_HUMAN	LLQNGNSPAEVAK
2	NCOA3_HUMAN	AVSLDSPVSVGSSPPVK
2	NCOAT_HUMAN	EIPVESIEEVSK
2	NEK2_HUMAN	SQDSSPVLSELK
2	NEK2_HUMAN	YSDELNEIITR
2	NFKB1_HUMAN	LSPAPSK
2	OGT1_HUMAN	GSVAEAEDC YNTALR
2	OGT1_HUMAN	AFLDSLPDVK
2	PCNA_HUMAN	FSASGELGNGNIK
2	PCNA_HUMAN	DLSHIGDAVVISC AK
2	PCP4_HUMAN	AAVAIQSQFR
2	PLK1_HUMAN	HINPVAASLIQK
2	RBP10_HUMAN	VQGTVHC FPISAR
2	RBP10_HUMAN	LYPAVNQQETPLPR
2	SKP2_HUMAN	LSDPIVNTLAK
2	SMAD4_HUMAN	GFPHVIYAR
2	SMAD4_HUMAN	YC QYAFDLK
2	STA5A_HUMAN	HILYNEQR
2	STA5A_HUMAN	ATIISEQQAK
2	STAT1_HUMAN	NLSFFLTPPC AR
2	STAT2_HUMAN	HYQLLTEENIPENPLR
2	STAT2_HUMAN	EVLQSLPLTEIIR
2	STAT3_HUMAN	FPELNYQLK
2	STAT3_HUMAN	GLSIEQLTTLAEK
2	STAT4_HUMAN	YLYPDIPK
2	VAV_HUMAN	YC SQVESASK
2	VAV_HUMAN	LNPGDIVELTK
