# Supplementary material for: Targeted proteomic assays for quantitation of proteins identified by proteogenomic analysis of ovarian cancer
Source: Sci Data. 2017 Jul 19;4:170091. doi: 10.1038/sdata.2017.91 (PMC5516542; doi:10.1038/sdata.2017.91)
Supplement: Supplementary Information [file sdata201791-s2.pdf]

Supplementary Figures

Targeted proteomics assays for quantitation of proteins identified by proteogenomic analysis of ovarian cancer

Ehwang Song[1,*], Yuqian Gao[1,*], Chaochao Wu[1], Tujin Shi[1], Song Nie[1], Thomas L. Fillmore[2], Athena A. Schepmoes[1], Marina A. Gritsenko[1], Wei-Jun Qian[1], Richard D. Smith[1], Karin D. Rodland[1], and Tao Liu[1,#]

[1]Biological Sciences Division and [2]Environmental Molecular Sciences Laboratory, Pacific Northwest National Laboratory, Richland, WA

*Co-first author

#Correspondence Author:
Tao Liu, Ph.D.
Phone: 509-371-6346
Fax: 509-371-6564
Email: tao.liu@pnnl.gov

Figure legends

- **Supplementary Figure 1**: Overview of SRM Assay Development and Validation. Initial list of target peptides contained 353 peptides selected based on the selection criteria (see Methods). Typically, 5 peptides were chosen for each protein and the crude peptides were synthesized. After optimizing SRM parameters and checking LC and MS behaviors of the initial target peptides, 145 peptides (~2 peptides/protein) were retained and the pure peptides were purchased. The 145 selected peptides (available in Supplementary Table 1a) were subjected to Experiment 1 to create the response curve. In this step, ~30% of the peptides were excluded from further testing because they did not meet certain specific requirement(s) of the CPTAC Assay Portal (e.g., dynamic range, precision, interference). The finalized list contains 98 assays which were validated in Experiments 1 to 4; 37 of the 98 assays also led to endogenous detection in Experiment 5. A separate PRISM-SRM analysis was also performed to assess the endogenous levels of the proteins in the tissue matrix tested, which detected 28 additional peptides.
- **Supplementary Figure 2**: Details of the Experiment 4 results. Peak areas of light (a) and heavy (b) peptides as well as the peak area ratios (c) from Batch 1 are depicted; the error bars represent SD values from the different storage time and conditions. The same results for peptides from Batch 2 are shown in d, e, and f, respectively. Peptides with much higher peak areas or ratios are shown in the insets.
- **Supplementary Figure 3**: Correlation of ELISA and LC-SRM results for quantification of PCNA. The SRM quantification of peptide DLSHIGDAVVISCAK showed good correlation to the ELISA detection of PCNA ($R^2 = 0.9627$) in 6 ovarian tissue samples. The stages of the 6 ovarian tissues samples are shown in the inset table. The control samples are not diagnosed with ovarian cancer.

Supplementary Figure 1. Overview of SRM Assay Development and Validation

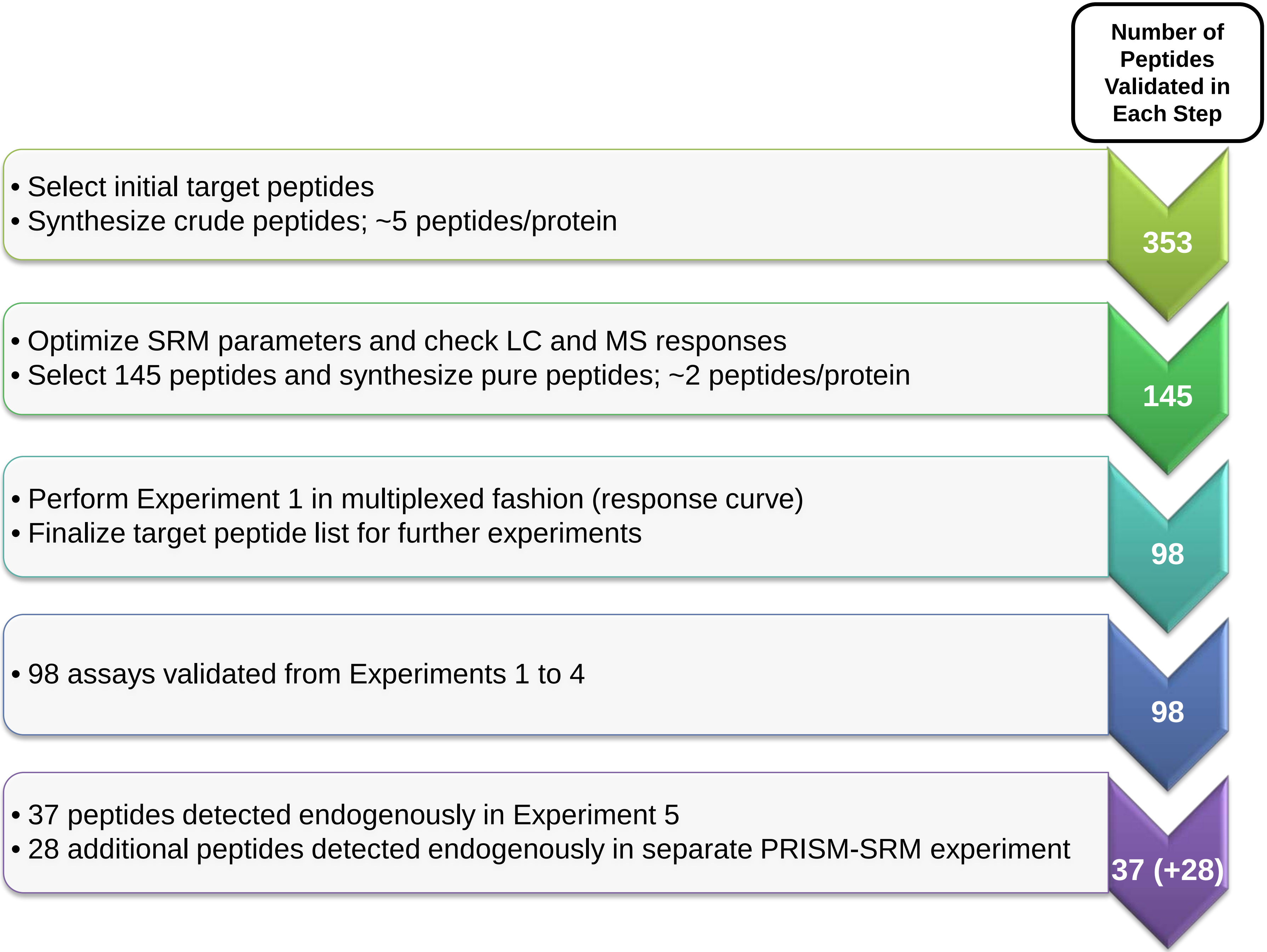

Supplementary Figure 2a. Peak Areas of Light Peptides from Batch 1

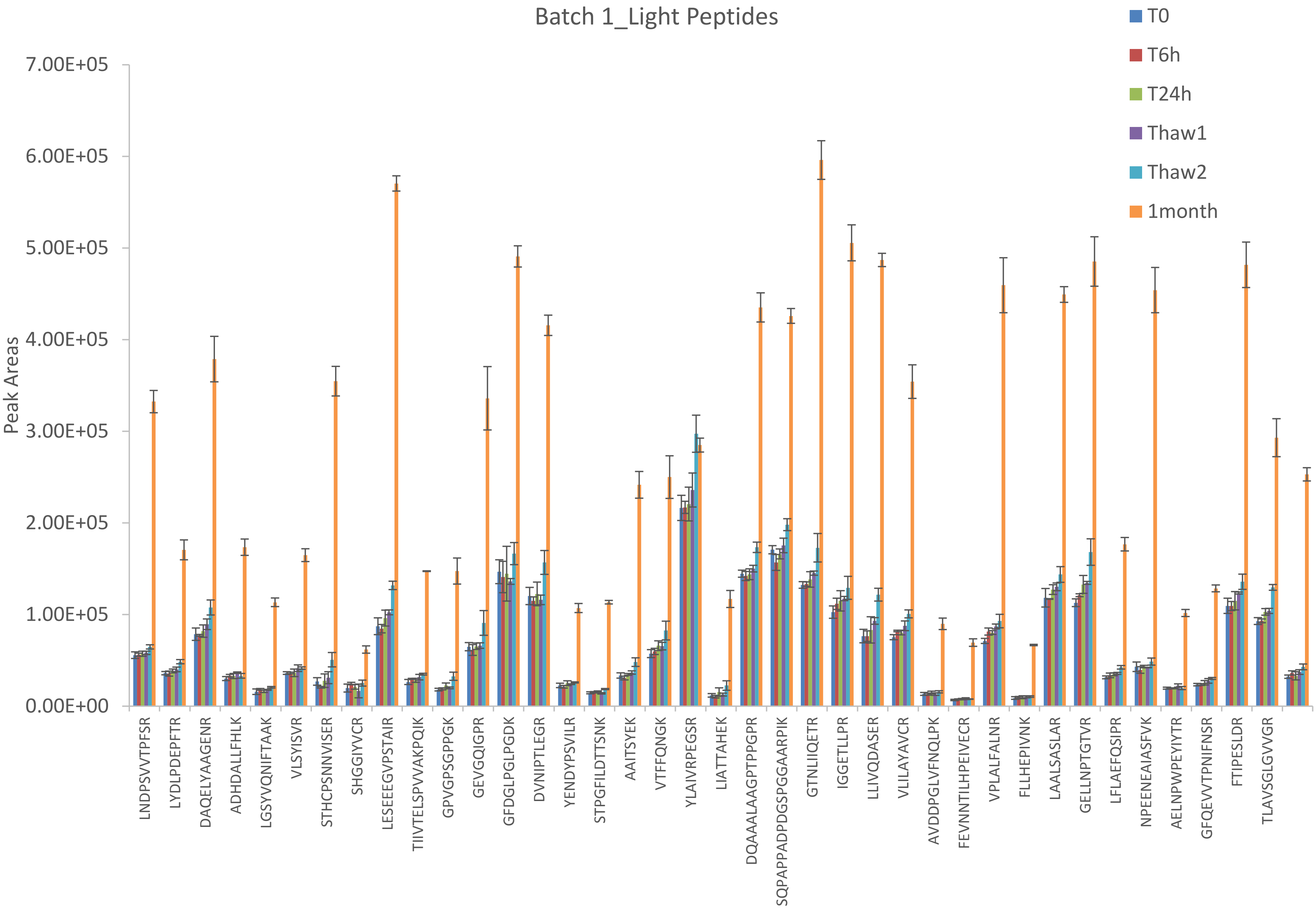

Supplementary Figure 2b. Peak Areas of Heavy Peptides from Batch 1

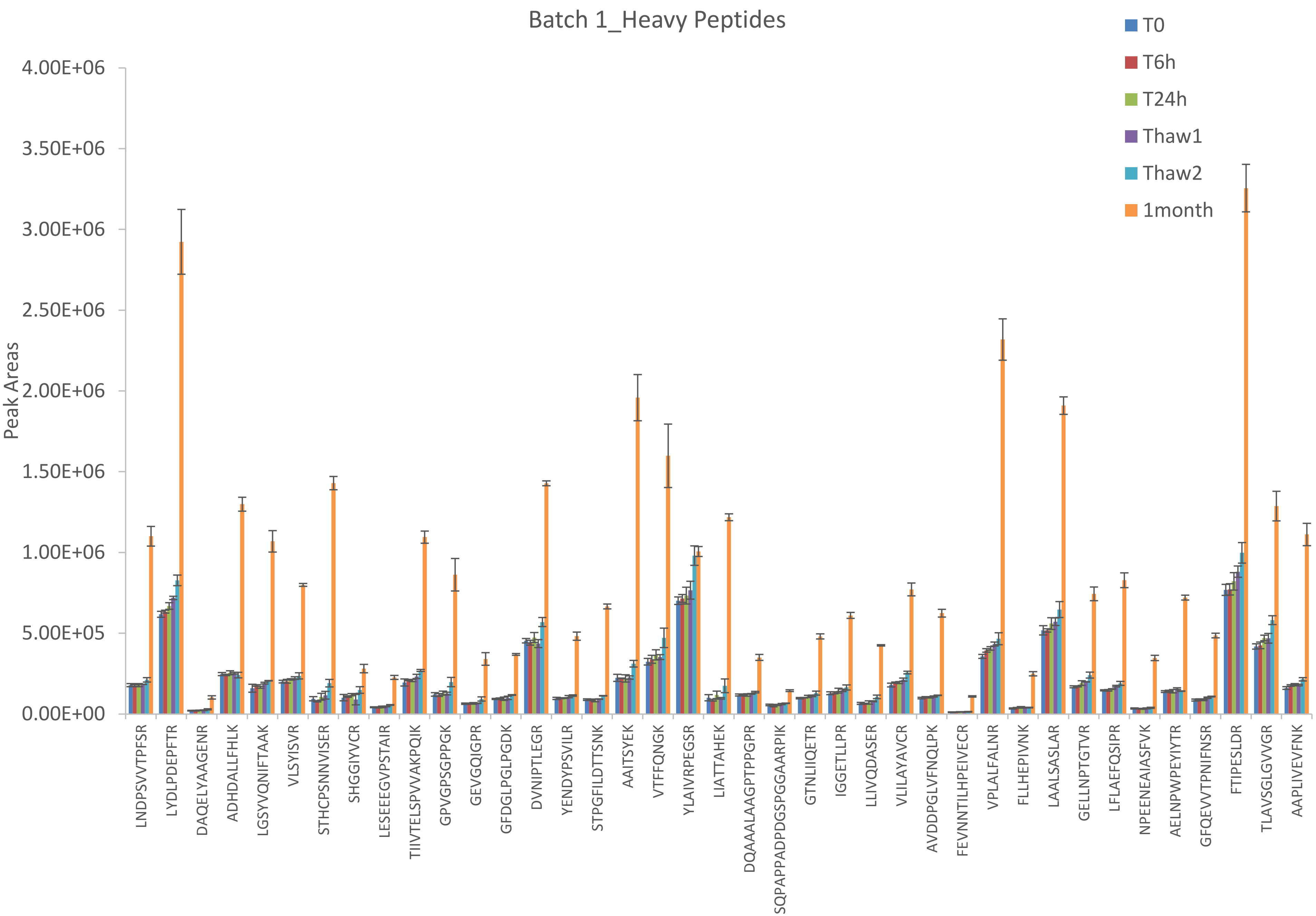

Supplementary Figure 2c. Peak Area Ratios (H/L) of Batch 1 Assays

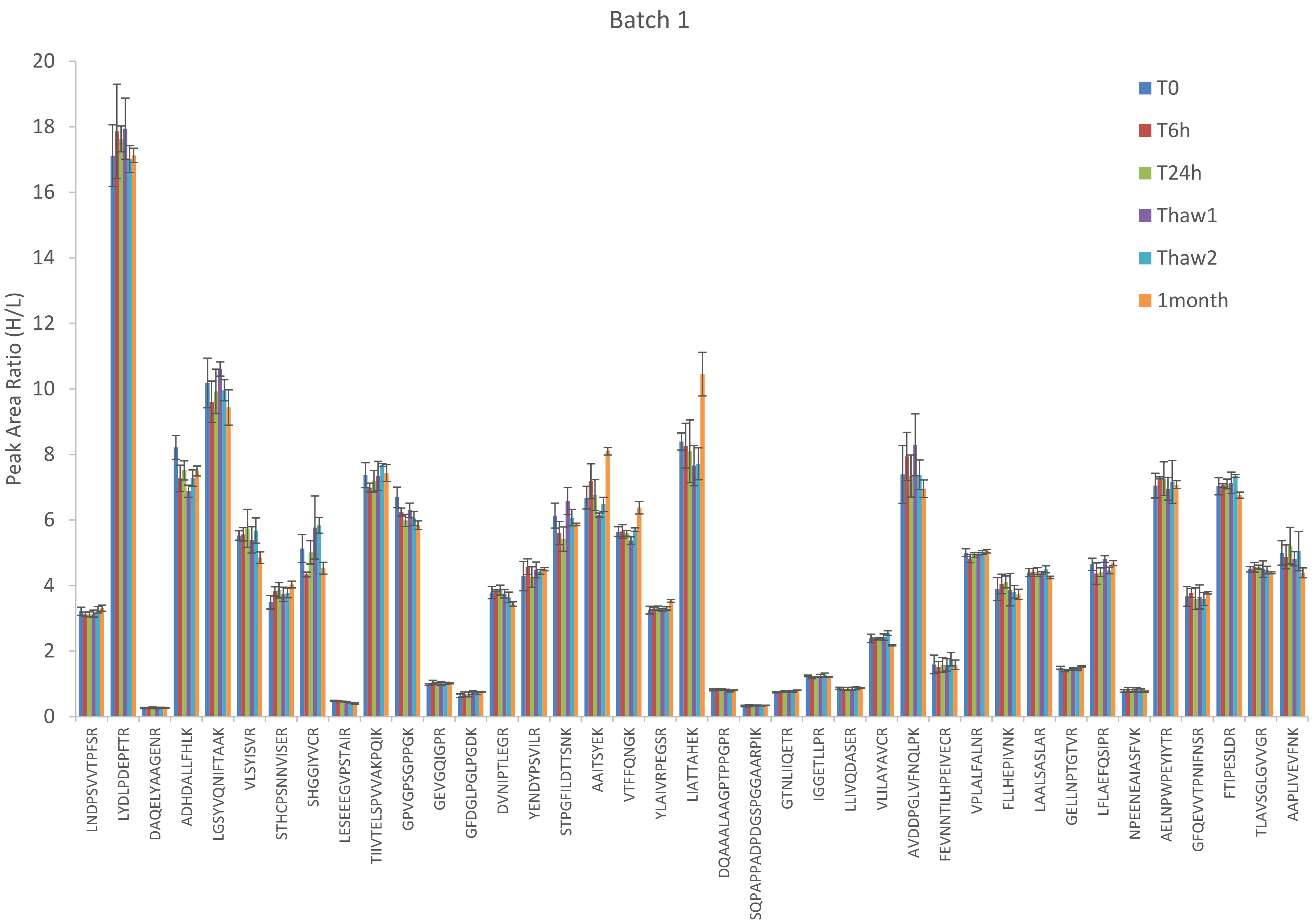

Supplementary Figure 2d. Peak Areas of Light Peptides from Batch 2

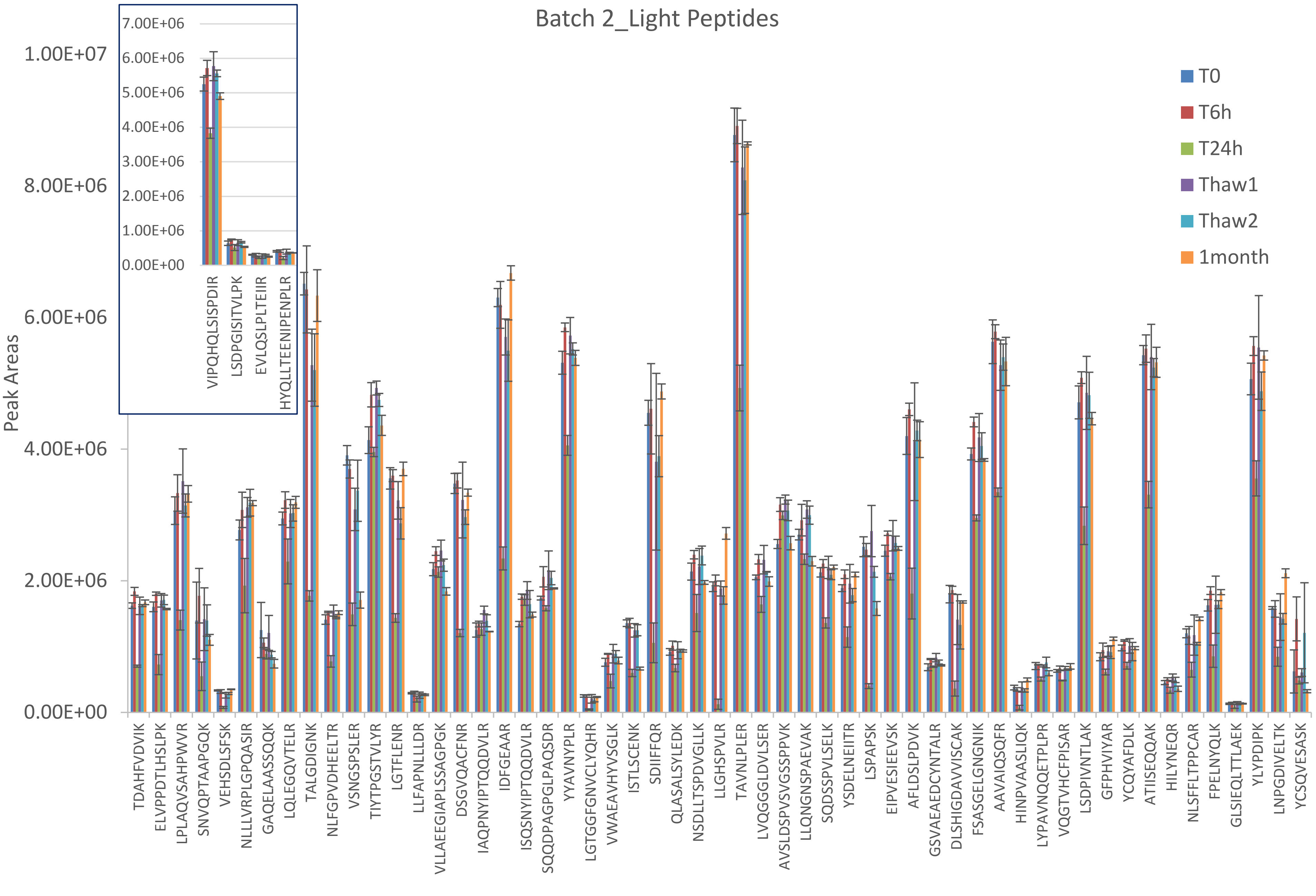

Supplementary Figure 2e. Peak Areas of Heavy Peptides from Batch 2

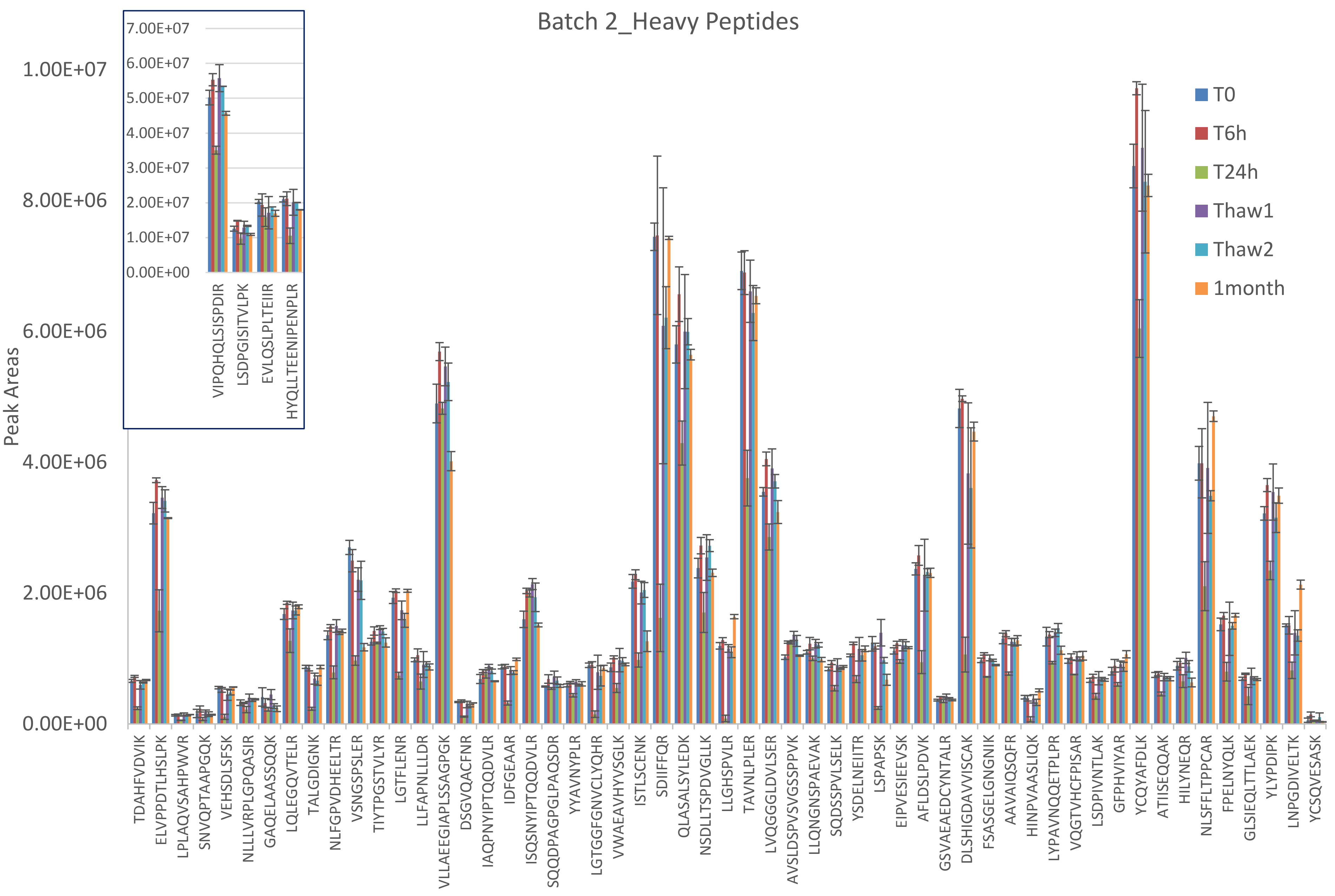

Supplementary Figure 2f. Peak Area Ratios (H/L) of Batch 2 Assays

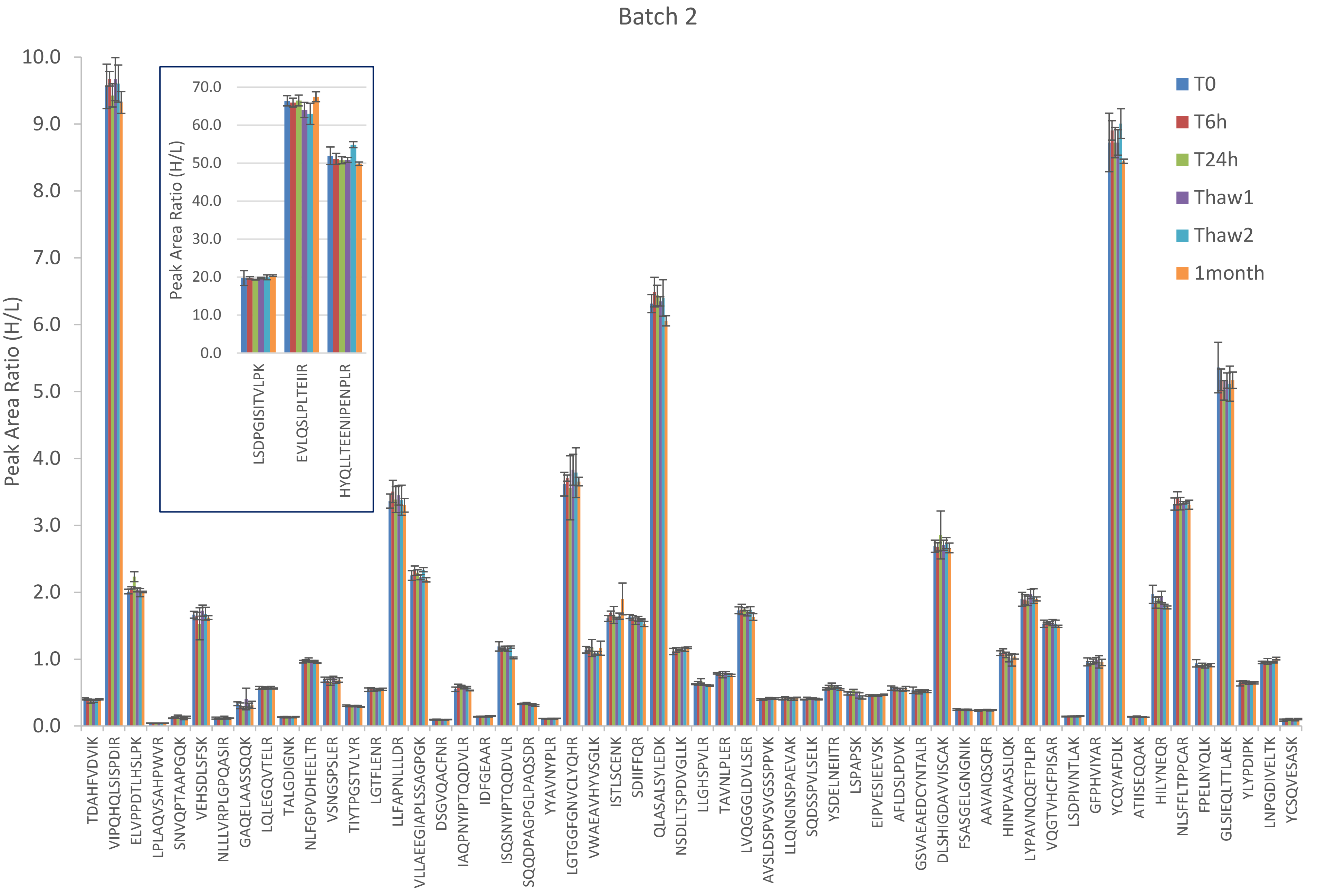

Supplementary Figure 3. Correlation of ELISA and LC-SRM results for quantification of PCNA

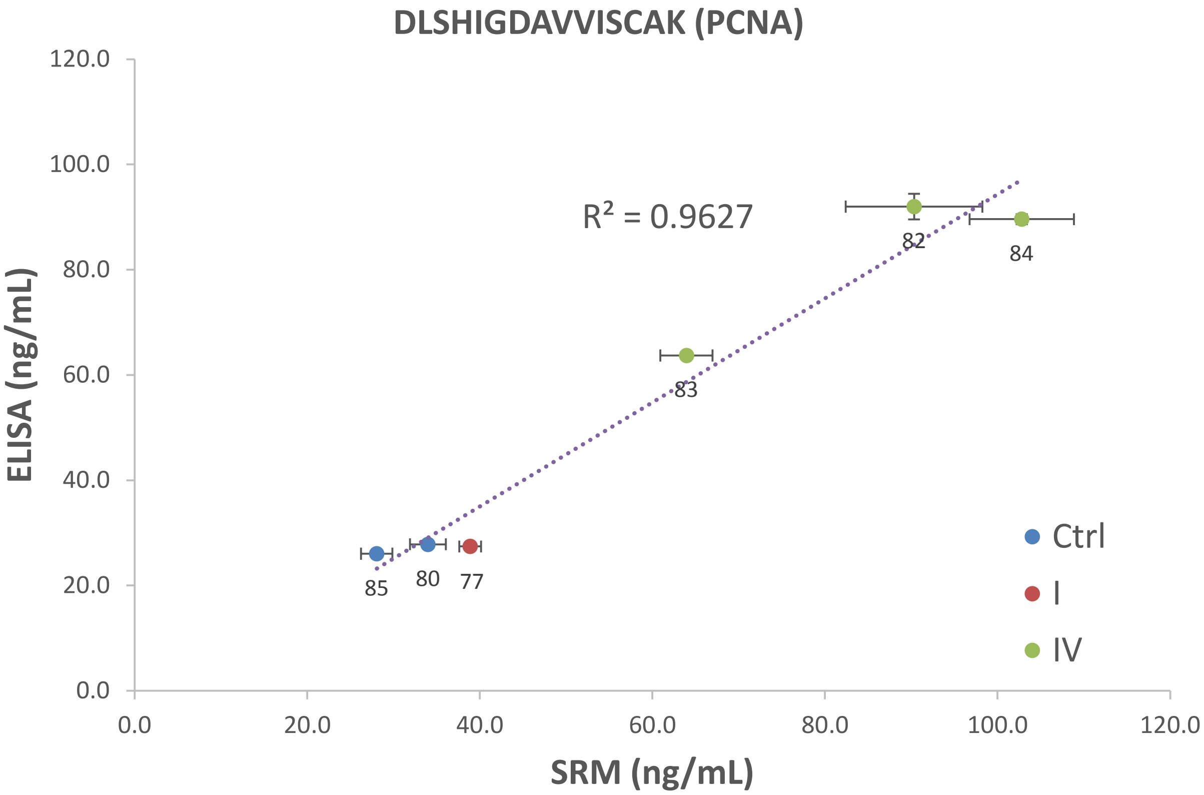

Sample	Stage
80	Ctrl
85	Ctrl
77	I
82	IV
83	IV
84	IV
